# New‐onset posttransplant diabetes mellitus after haploidentical hematopoietic cell transplantation with posttransplant cyclophosphamide

**DOI:** 10.1002/jha2.70

**Published:** 2020-09-23

**Authors:** Brendan L. Mangan, Dilan Patel, Heidi Chen, Katie S. Gatwood, Michael T. Byrne, Salyka Sengsayadeth, Stacey Goodman, Bhagirathbhai Dholaria, Adetola A. Kassim, Madan Jagasia, Wichai Chinratanalab, Kathryn A. Culos, Brian G. Engelhardt

**Affiliations:** ^1^ Department of Pharmacy Vanderbilt University Medical Center Nashville Tennessee; ^2^ Division of Hematology and Oncology Department of Medicine Vanderbilt University Medical Center Nashville Tennessee; ^3^ Department of Biostatistics Vanderbilt University Medical Center Nashville Tennessee

**Keywords:** haploidentical, hematopoietic cell transplant, posttransplant diabetes mellitus

## Abstract

Haploidentical hematopoietic cell transplantation (haplo‐HCT) with posttransplant cyclophosphamide (PTCY) is utilized for patients with hematological disorders but without conventional donors. The effects of new‐onset posttransplant diabetes mellitus (PTDM) following haplo‐HCT are unknown. We examined PTDM incidence and outcomes after haplo‐HCT with PTCY. Patients without diabetes receiving haplo‐HCT (n = 64) were analyzed for PTDM diagnosis (defined as blood glucose ≥ 200 mg/dL). By day 100, 14 (22%) patients developed PTDM (median, 18 days). Hyperglycemia (blood glucose ≥ 200 mg/dL) preceded corticosteroids in 11 (79%) individuals. PTDM patients had increased death/relapse (*P* = .029). PTDM occurs frequently, precedes corticosteroids, and leads to inferior outcomes following haplo‐HCT. PTDM prophylaxis/treatment may improve HCT survival.

## 1 BACKGROUND

New‐onset posttransplant diabetes mellitus (PTDM) occurs in 20‐60% of patients undergoing allogeneic hematopoietic cell transplantation (HCT) with traditional conditioning regimens but without posttransplant cyclophosphamide (PTCY).[1‐4] Both pre‐existing diabetes mellitus and new‐onset PTDM have been shown to decrease survival after conventional HLA‐matched related or unrelated donor HCT [1, 2, 5, 6]. The incidence and outcomes of patients developing PTDM after haplo‐identical transplant (haplo‐HCT) with PTCY are unknown.

Growing numbers of patients requiring transplant but without a conventional related or unrelated donor are undergoing HCT using a haploidentical first‐degree relative with at least five of 10 HLA match between the recipient and donor. [7, 8] Due to intense bidirectional alloreactivity, many patients receive PTCY on days 3 and 4 after transplant to prevent graft rejection and graft‐versus‐host disease (GVHD) [9]. By targeting activated T cells and by promoting regulatory T‐cell (Treg) reconstitution, immune tolerance can be achieved with PTCY [6, 9]. In general, diabetes mellitus is an inflammatory condition characterized by increased Th1 immunity and lower frequencies of Tregs [10]. By increasing or decreasing immune activation, HLA mismatch or PTCY could either promote or suppress PTDM development after haplo‐HCT, respectively. As previously discussed, PTDM in HLA‐matched HCTs has a negative effect on survival, though a lack of data exists for the distinct haplo‐HCT population. Therefore, we examined the incidence, risk factors, and outcomes for PTDM after haplo‐HCT with PTCY. We hypothesized that hyperglycemia would occur despite PTCY and that PTDM would affect survival negatively following haplo‐HCT.

## 2 METHODS

A single‐center, retrospective study was conducted at Vanderbilt University Medical Center (VUMC) to assess the incidence, clinical risk factors, and associated outcomes of PTDM in patients without established diabetes mellitus undergoing first haplo‐HCT. The Vanderbilt institutional review board approved the methods.

From January 1, 2013 through July 31, 2018, pediatric and adult patients who received an ablative or reduced intensity haplo‐HCT for malignant or nonmalignant diseases at VUMC were eligible. Patients received GVHD prophylaxis with PTCY, mycophenolate mofetil until day 30‐40, and either tacrolimus or sirolimus. Two pediatric patients with immunodeficiency disorders did not receive GVHD prophylaxis. Exclusion criteria included nonhaplo‐HCTs, second HCT, or pre‐existing diabetes mellitus as determined by self‐reported history or the presence of insulin/diabetes medication on their medication list.

The primary objective was the incidence of PTDM by day 100. New‐onset PTDM was defined as any random blood glucose ≥ 200 mg/dL. Secondary outcomes included overall survival (OS), disease‐free survival (DFS), nonrelapse mortality, cumulative incidence of malignancy relapse, incidence of grade 2‐4 acute GVHD, time to systemic glucocorticoids, and risk factors for PTDM [11]. The statistical analysis plan is discussed in the Supporting Information Methods.

## 3 RESULTS

From January 1, 2013 to July 31, 2018, 80 patients underwent haplo‐HCT with PTCY. Sixteen patients were excluded due to pre‐existing diabetes prior to transplant (n = 12) or for receiving second allogeneic HCT (n = 4). Clinical characteristics for the remaining 64 haplo‐HCT recipients are presented in Table 1.

**TABLE 1:** Characteristics of patients undergoing haplo‐identical hematopoietic cell transplant (haplo‐HCT), stratified for posttransplant diabetes mellitus (PTDM) development

Characteristic	PTDM, n = 14 (%)	No PTDM, n = 50 (%)	*P*‐value
Median age, years (range)	37(0.9‐71.5)	44 (0.1‐71.9)	.500
Male	8 (57)	32 (64)	.639
Caucasian	8 (57)	30 (60)	.609
BMI (range)	23.7 (14.2‐37.5)	25.7 (11.9‐45.7)	.700
Hypertension	2 (14)	10 (20)	.628
Disease^a^			
Myeloid	9 (64)	24 (48)	.554
Lymphoid	1 (7)	6 (12)	
Nonmalignant	4 (29)	20 (40)	
Modified disease risk index^b^			
Very low risk	4 (29)	20 (40)	.141
Low	3 (21)	3 (6)	
Intermediate	2 (14)	13 (26)	
High	2 (14)	11 (22)	
Very high	3 (21)	3 (6)	
Chemotherapy			
Myeloablative	7 (50)	16 (32)	.215
Reduced intensity	7 (50)	34 (68)	
Thymoglobulin	3 (21)	10 (20)	.934
Stem cell source			
Peripheral blood	11 (79)	34 68)	.444
Bone marrow	3 (21)	16 (32)	
GVHD prophylaxis			
FK/MMF	12 (86)	35 (70)	.475
Sirolimus/MMF	2 (14)	13 (26)	
None	0 (0)	2 (4)	
Time to PTDM, days (range)	18 (8‐72)	–	N/A
Grade 2‐4 GVHD	6 (43)	12 (24)	.165
Time to grade 2‐4 GVHD, days (range)	37 (14‐79)	37 (16‐95)	.196
Steroid treatment^c^	7 (50)	14 (28)	.121
Time to steroids, days (range)	40 (17‐90)	39 (0‐95)	.492
Max prednisone dose, mg/kg/day (range)	0.48 (0.07‐1.21)	0.44 (0.07‐1.67)	.829

Abbreviations: ALL, acute lymphoblastic leukemia; AML, acute myelogenous leukemia; BMI, body mass index; CML, chronic myelogenous leukemia; FK, tacrolimus; GVHD, graft‐versus‐host‐disease; MDS, myelodysplastic syndrome; MMF, mycophenolate mofetil; MPN, myeloproliferative neoplasm; NHL, non‐Hodgkin's lymphoma; PTCY, posttransplant cyclophosphamide; PTDM, posttransplant diabetes mellitus.

a Malignant diseases included AML (n = 20), MDS (n = 9), CML (n = 4), ALL (n = 1), NHL (n = 3), myelofibrosis (n = 2), and Hodgkin's lymphoma (n = 1). Nonmalignant diseases included sickle cell anemia (n = 12), aplastic anemia (n = 4), severe combined immunodeficiency (n = 3), beta thalassemia (n = 2), Huntington disease (n = 2), and Fanconi's anemia (n = 1).

b Disease risk index was modified to include a very low risk category for nonmalignant diseases.

c Indications for steroid treatment: GVHD (n = 17), adrenal insufficiency (n = 2), drug rash (n = 1), and arthritis (n = 1).

**FIGURE 1:**
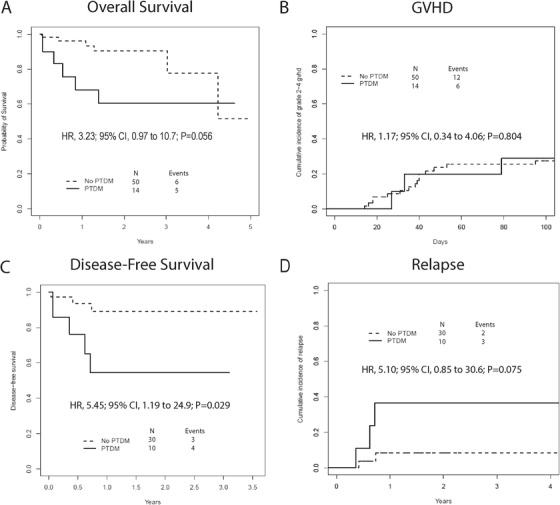
Haplo‐identical hematopoietic cell transplant (haplo‐HCT) outcomes stratified for development of posttransplant diabetes mellitus (PTDM). Overall survival and cumulative incidence of acute graft‐versus‐host‐disease (GVHD) for the entire cohort (panels A and B, respectively). Disease‐free survival and cumulative incidence of relapse for patients with malignancy only (panels C and D, respectively)

The primary outcome of new‐onset PTDM was met in 14 patients (22%), diagnosed at a median of 18 days after transplant (range, 8‐72 days). Hyperglycemia (blood glucose ≥ 200 mg/dL) preceded grade 2‐4 GVHD and corticosteroids in 10 (71%) and 11 (79%) individuals, respectively. The day 100 cumulative incidence of grade 2‐4 GVHD was low after haplo‐HCT with PTCY and was not statistically different between the PTDM and non‐PTDM groups (29%; 95% confidence interval [CI], 0‐52 vs 27%; 95% CI, 15‐38; *P* = .804) (Figure 1). Other clinical characteristics were also similar between the PTDM and euglycemic cohorts (Table 1). Since tissue injury/inflammation may contribute to hyperglycemia after transplant, chemotherapy intensity and alloreactivity were selected a priori as variables of interest. In multivariate logistic regression, neither ablative conditioning (odds ratio [OR], 4.02; 95% CI, 0.90‐17.99; *P* = .068] nor grade 2‐4 GVHD (OR, 0.92; 95% CI, 0.11‐ 7.63; *P* = .936) predicted PTDM diagnosis.

Among surviving patients (n = 53), median follow‐up was 1.74 years (range, 0.29‐5 years). Malignancy relapse/progression occurred in five HCT recipients and resulted in five deaths. Other causes of mortality included graft failure (n = 3), infection (n = 2), and GVHD (n = 1) (Figure S1). Although we did not observe excess cardiovascular mortality in our hyperglycemic patients, OS at 1 year tended to be lower in patients developing PTDM compared to non‐PTDM individuals (68%; 95% CI, 47‐99 vs 96%; 95% CI, 91‐100; hazard ratio [HR], 3.23; 95% CI, 0.97‐10.74; *P* = .056). The inferior outcomes for PTDM patients appeared to be partly related to increased risk for malignancy relapse (HR, 5.1; 95% CI, 0.85‐30.6; *P* = .075) leading to lower DFS (HR, 5.45; 95% CI, 1.19‐24.9; *P* = .029) (Figure 1). Since malignancy relapse and glycemia status appeared to be important drivers of survival, multivariable models were constructed. After adjustment for the DRI score, PTDM still appeared to be associated with increased risk of death or relapse (HR, 4.81; 95% CI, 1.02‐22.7; *P* = .048) (Table S1) [17].

## 4 DISCUSSION

PTDM developed in about one quarter of patients undergoing haplo‐HCT and increased the risk of death or relapse four times. These data are novel since previous PTDM research focused on conventional donor HCT and the incidence of hyperglycemia after PTCY is not well studied. As in other research, hyperglycemia generally preceded the initiation of corticosteroids [12]. This is important because it negates the commonly held belief that PTDM is merely a side effect of immunosuppression. Although it is possible that corticosteroids exacerbate clinical hyperglycemia, our data indicate that glucose metabolism is already altered by the time these medications are started and other causes for PTDM need to be identified.

From previous trials, the incidence of PTDM has ranged from about 20 to 60% in the nonhaplo‐HCT population [1, 2, 3, 4]. The 22% development of PTDM in this study falls within that range, but on the lower side. The lower incidence after haplo‐HCT could be due to the lack of routine fasting labs. In this retrospective study, episodes of fasting hyperglycemia could have been missed leading to an underestimate of the true rate of PTDM. Another hypothesis to account for this lower incidence may be the conditioning regimen and PTCY used in the haplo‐HCT population. Cyclophosphamide selectively depletes alloreactive T cells sparing T cells responsible for immune reconstitution and regulation [9]. Post‐HCT cyclophosphamide given on days 3 and 4 after haplo‐HCT has been shown to be an effective method for GVHD prophylaxis. Diabetes mellitus has a known inflammatory component linked with insulin resistance due to activated T lymphocytes and increased inflammatory cytokines [10, 12]. In turn, cyclophosphamide may play a role in decreasing inflammation, leading to lower rates of PTDM after haplo‐HCT. Although further research is required to validate, our proposed model of inflammation‐induced PTDM is outlined in Figure S2.

Older age, nonwhite ethnicity, corticosteroids, parenteral nutrition, unrelated donor, ablative conditioning, impaired fasting glucose, and elevated C‐peptide levels have been reported as risk factors for PTDM following HCT with conventional HLA‐matched donors.[3, 4] In our study, ablative chemotherapy and steroid exposure were more common in the PTDM group, however this did not reach statistical significance. We were unable to definitively identify any clinical predictors of PTDM after haplo‐HCT. It is possible that previously reported risk factors for PTDM are not as relevant following PTCY. As previously mentioned, our research was limited by its retrospective nature, lack of routine fasting blood glucose measurements, and modest cohort size that may have influenced the results. It is likely that we did not have enough statistical power to detect differences between the PTDM and non‐PTDM groups. In addition, the multivariate regression model may have been over fit for the cohort size.

Diabetes is known to complicate cancer care. The HCT comorbidity index has shown that pre‐existing diabetes mellitus increases mortality after HCT [13]. In this context, we were not surprised by the inferior outcomes for patients developing new‐onset PTDM after haplo‐HCT. Interestingly, we also noted a trend for excess relapse in PTDM patients. This finding may not be coincidental as large epidemiological studies have shown a strong bidirectional association between cancer and diabetes diagnoses [14]. From a biological perspective, mutations involved with leukemogenesis (DNMT3A, TET2, and ASXL1) are also enriched in patients with diabetes and atherosclerotic disease but without cancer. The presence of shared mutations that contribute to both myeloid malignancy and glucose dysregulation may partly explain the relationship between diabetes and cancer or relapse after HCT [15]. Much work still needs to be done to elucidate the biological pathways linking diabetes to cancer. Unfortunately, incomplete data precluded mutational analysis in our haplo‐HCT cohort.

Despite receiving immunomodulatory PTCY, 22% of haplo‐HCT recipients developed new‐onset PTDM by day 100. The development of PTDM was independent of corticosteroids and was associated with a decrease in OS and DFS. At least two other prospective and several retrospective studies have confirmed the high incidence and negative consequences of PTDM after HCT (Table S2) [1, 2, 5, 6, 12, 16]. With the clinical importance now established, it is time to shift focus from examining the incidence of PTDM, and begin to study the mechanistic causes for hyperglycemia and potential therapeutic options to prevent or treat metabolic complications after HCT. Understanding the actionable targets that lead to PTDM remains an unmet need and should be actively investigated to optimize patient outcomes.

## AUTHOR CONTRIBUTIONS

Brendan Mangan, Dilan Patel, Kathryn A. Culos, and Brian G. Engelhardt designed research, collected data, analyzed and interpreted data, wrote the manuscript, and revised the manuscript. Heidi Chen performed statistical analysis and interpreted data. Katie S. Gatwood, Michael T. Byrne, Salyka Sengsayadeth, Stacey Goodman, Bhagirathbhai Dholaria, Adetola A. Kassim, Madan Jagasia, and Wichai Chinratanalab analyzed and interpreted data, revised manuscript.

## CONFLICT OF INTEREST

The authors declare no conflict of interest.

5

## Supporting information

Supporting Information.

Supporting Information.

Supporting Information.

## Data Availability

The data that support the findings of this study are available from the corresponding author upon reasonable request.
