# Supplementary material for: New‐onset posttransplant diabetes mellitus after haploidentical hematopoietic cell transplantation with posttransplant cyclophosphamide
Source: EJHaem. 2020 Sep 23;1(2):576–80. doi: 10.1002/jha2.70 (PMC7942195; doi:10.1002/jha2.70)
Supplement: Supplementary file 1 — Supporting Information. [file JHA2-1-576-s002.pdf]

Supplemental Figure 1

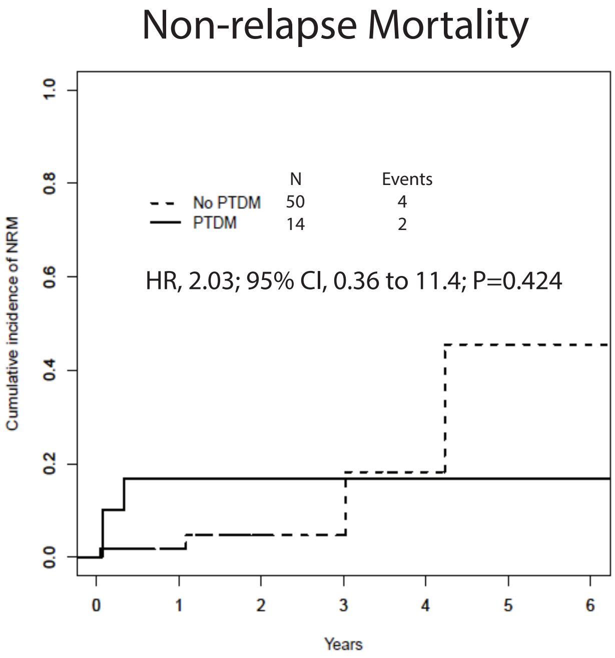

Supplemental Figure 1. Cumulative incidence of non-relapse mortality following haploidentical hematopoietic cell transplant (Haplo-HCT) stratified for the development of post-transplant diabetes mellitus (PTDM).
