# Supplementary material for: New‐onset posttransplant diabetes mellitus after haploidentical hematopoietic cell transplantation with posttransplant cyclophosphamide
Source: EJHaem. 2020 Sep 23;1(2):576–80. doi: 10.1002/jha2.70 (PMC7942195; doi:10.1002/jha2.70)
Supplement: Supplementary file 2 — Supporting Information. [file JHA2-1-576-s003.pptx]

## Slide 1
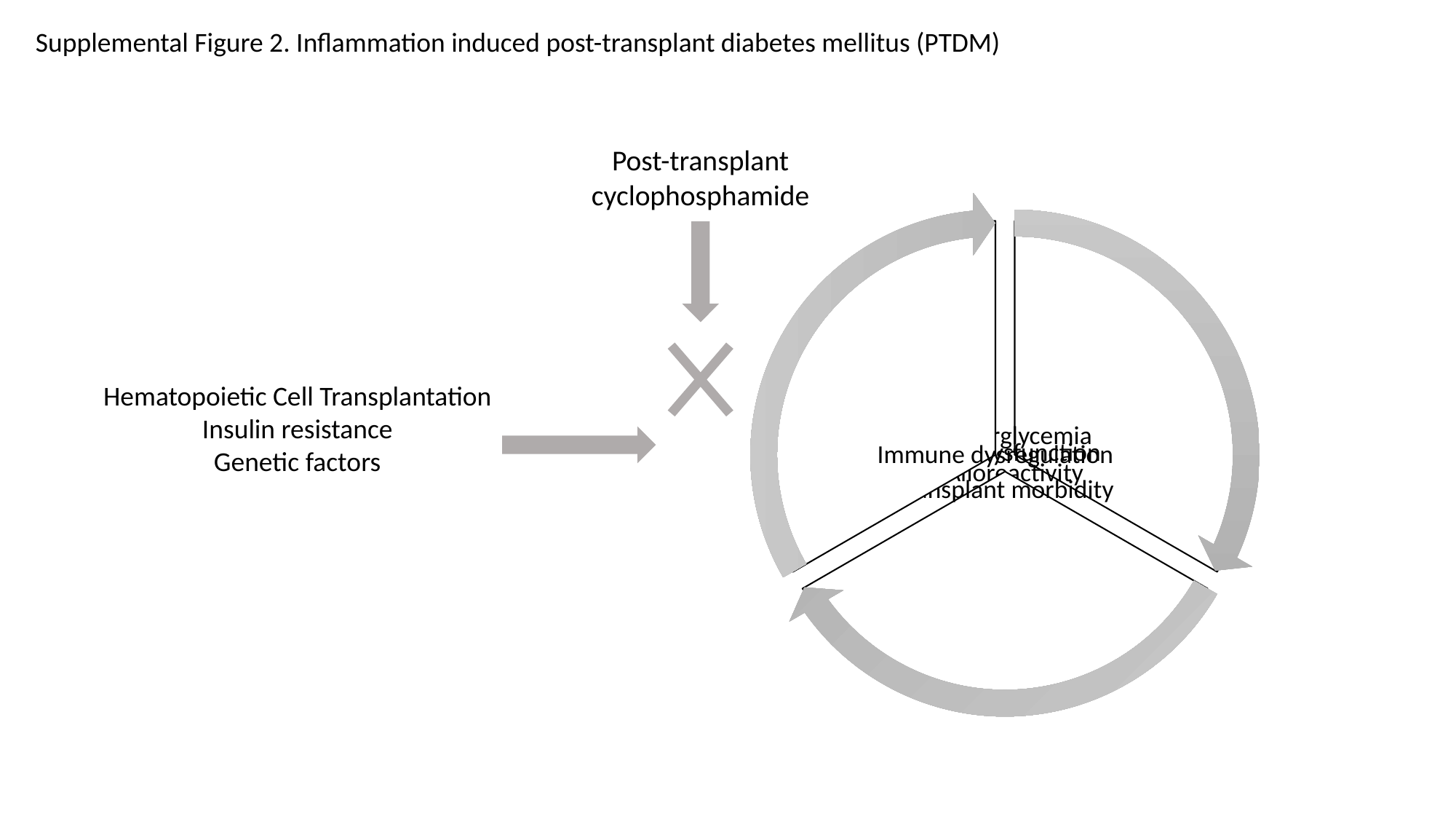

Supplemental Figure 2. Inflammation induced post-transplant diabetes mellitus (PTDM)
Post-transplant cyclophosphamide
Hematopoietic Cell Transplantation
Insulin resistance
Genetic factors
