# Supplementary material for: New‐onset posttransplant diabetes mellitus after haploidentical hematopoietic cell transplantation with posttransplant cyclophosphamide
Source: EJHaem. 2020 Sep 23;1(2):576–80. doi: 10.1002/jha2.70 (PMC7942195; doi:10.1002/jha2.70)
Supplement: Supplementary file 3 — Supporting Information. [file JHA2-1-576-s001.docx]

**Supplemental Methods**

**Statistics**

Descriptive statistics including median and range for continuous variables as well as percent and frequencies for categorical variables were presented. Between group comparisons were performed using chi square and Mann-Whitney U test for categorical and continuous variables, respectively. Continuous variables between 3 or more groups were analyzed with one-way ANOVA. Multivariable logistic regression was applied to identify risk factors associated with the development of PTDM. Survival curves were calculated from the Kaplan-Meier method for the PTDM groups and were compared using the log rank test, Cox proportional hazards regression, or Fine and Gray method. PTDM diagnosis was treated as a time-dependent variable in order to remove immortal time bias. Multivariable survival models were constructed using PTDM and a modified disease risk index (DRI) score.([17](#_ENREF_17)) The DRI was adapted to include a very low risk category for non-malignant diseases. Data was analyzed with R version 3.3.1 (R Foundation for Statistical Computing, Vienna, Austria). Statistical tests were 2-sided and considered significant at P ≤ 0.05.

**Supplemental Figures**

Supplemental Figure 1: Cumulative incidence of non-relapse mortality following haploidentical hematopoietic cell transplant (haplo-HCT) stratified for development of post-transplant diabetes mellitus (PTDM).

Supplemental Figure 2: Inflammation induced post-transplant diabetes mellitus (PTDM)

**Supplemental Tables**

Supplemental Table 1. Multivariate analysis of overall survival (OS) for the entire cohort or disease-free survival (DFS) for patients with malignancy following haplo-identical hematopoietic cell transplant (haplo-HCT).

**Outcome Variable Hazzard ratio 95% Confidence interval P-value**

OS PTDM 2.82 0.83 to 9.59 0.096

DRI 1.44 0.94 to 2.21 0.095

DFS PTDM 4.81 1.02 to 22.7 0.048

DRI 2.06 0.93 to 4.58 0.074

OS, overall survival; DFS, disease-free survival; PTDM, post-transplant diabetes mellitus; DRI, disease risk index.

Supplemental Table 2. Summary of survival outcomes for patients developing new-onset post-transplant diabetes mellitus (PTDM) after hematopoietic cell transplant (HCT)

| **Patient population** | **HCT type** | **Methods** | **Day 100 PTDM incidence** | **PTDM/HCT Outcome** | **References** |
| --- | --- | --- | --- | --- | --- |
| Adult | MRD+URD | Retrospective | Unknown  (malglycemia^A^) | Increased non-relapse mortality | Hammer *et. al.* 2009([5](#_ENREF_5)) |
| Adult | MRD+URD | Prospective | 60% | Decreased survival | Griffith *et. al*.  2011([2](#_ENREF_2)) |
| Adult | MRD+URD | Prospective + retrospective | Cohort 1: 67%  Cohort 2: 58% | Increased non-relapse mortality | Johnpulle *et. al.* 2016([12](#_ENREF_12)) |
| Pediatric | MRD+URD+  Autologous | Retrospective | Unknown  (44% with malglycemia^A^) | Decreased survival + increased non-relapse mortality | Sopfe *et. al.* 2019([16](#_ENREF_16)) |
| Adult | MRD | Prospective | 55% | Decreased survival | Engelhardt *et. al.* 2019([1](#_ENREF_1)) |
| Pediatric | MRD+URD+Cord | Prospective | 31% | Decreased survival | Rowan *et. al.* 2020([6](#_ENREF_6)) |

^A^Malglycemia was defined by a composite of hypoglycemia, hyperglycemia, and/or glycemic variability.

PTDM, post-transplant diabetes mellitus; HCT, hematopoietic cell transplant; MRD, matched related donor, URD, unrelated donor
